# Measuring situational music performance anxiety – the Polish adaptation of the Performance-related Questionnaire for Musicians

**DOI:** 10.3389/fpsyg.2025.1705837

**Published:** 2025-11-13

**Authors:** Julia Kaleńska-Rodzaj, Michał Kotyczka, Manfred Nusseck, Claudia Spahn

**Affiliations:** 1 Department of Psychology, The University of the National Education Commission, Krakow, Poland; 2 Katowice Faculty of Psychology, SWPS University of Social Sciences and Humanities, Katowice, Poland; 3 Freiburg Institute for Musicians’ Medicine, University of Music Freiburg, Medical Center and Faculty of Medicine – University of Freiburg, Freiburg Center for Research and Teaching in Music, Freiburg, Germany

**Keywords:** music performance anxiety, live performance, polish adaptation, validation, factor analysis

## Abstract

**Introduction:**

Music performance anxiety (MPA) was mainly assessed using questionnaires that focus on a general predisposition to MPA, detached from a performance. Since performances vary greatly, an appropriate questionnaire should be used to investigate situation-specific MPA. The Performance-related Questionnaire for Musicians (PQM) is an instrument designed to measure situational MPA under live performance conditions. In this study, the Polish adaptation of the PQM has been developed and validated.

**Methods:**

Soloists, choral singers, and orchestral musicians (*N* = 258) completed a battery of self-report inventories immediately after their concert performance.

**Results:**

A confirmatory factor analysis (CFA) model with three factors of the dimensions Symptoms of MPA, Functional Coping, and Self-Efficacy across three time conditions before, during and after the performance showed optimal model fit and confirmed the stability of the factor structure in the original PQM. The reliability coefficients of the subscales were satisfactory, ranging from 0.63 to 0.87. Theoretical validity of the PQM subscales was also supported through correlations with the Performance Anxiety Inventory, Flow Short Scale, and General Self-Efficacy Scale.

**Discussion:**

The Polish adaptation of the PQM constitutes a reliable and valid tool for assessing situational MPA in adult musicians.

## 1 Introduction

Performing in the public eye often leads to changes in musicians’ emotional well-being that are not always predictable during the preparatory stage without an audience. The ability to regulate emotions effectively is crucial in the profession of a concert musician, as various emotions may arise from multiple sources: the emotional narrative within the musical piece, the performance setting (e.g., audience presence and social exposure), the uniqueness of the artist in terms of their personality (Spahn et al., 2024), previous performance experience, and typical behavior in performance situations (e.g., heightened self-awareness) (Spahn, 2015).

Research on emotions that arise in relation to a performance, especially pre-performance emotions, has adopted a variety of perspectives, represented along a continuum: positive emotions (e.g., enjoyment, self-confidence, Perdomo-Guevara, 2017, Perdomo-Guevara and Dibben, 2025); mixed emotions (e.g., a combination of enjoyment and anxiety, Kaleńska-Rodzaj, 2020, 2021; Osborne and McPherson, 2018; Lamont, 2012); facilitating or debilitating music performance anxiety (Wolfe, 1989; Murphy et al., 2024); and debilitating music performance anxiety (Kenny, 2011; Salmon, 1990). The first two perspectives, which encompass the full spectrum of emotional experiences, allow for both preventive actions – aimed at maintaining the musician’s emotional well-being, and intervention strategies – intended to strengthen self-efficacy and to help overcome emotional helplessness in the face of heightened mental and physiological anxiety responses. However, in performance practice, musicians themselves predominantly focus on the debilitating factors of music performance anxiety and wish to reduce these through interventions. Consequently, the latter two theoretical and empirical perspectives also frequently come to the fore in research as well. In most training formats for professional musicians today, however, a differentiated approach to music performance anxiety, including its positive aspects, is taught.

Music performance anxiety (MPA) is defined as an anxiety response of varying duration that arises in performance situations involving an audience. On a high level, this form of anxiety can interfere with the ability to deliver a satisfactory performance. In contrast, at moderate levels it can have a supportive and activating effect. The anxiety response is typically accompanied by physiological, cognitive, and behavioral symptoms (Spahn, 2015; Kenny, 2011; Papageorgi et al., 2007; Salmon, 1990). Research on MPA has primarily focused on understanding the underlying mechanisms of its development, as well as its impact on both performance quality and performers’ subjective satisfaction. Findings indicate that between 20 and 50% of professional musicians experience negative emotions associated with public performance (Kenny et al., 2004; Kenny et al., 2012; Paliaukiene et al., 2018). MPA is a widespread issue, affecting both amateur musicians—approximately 40% (Spahn et al., 2023; Burin et al., 2019)—and elite professionals, with prevalence rates reaching 56% (Osborne and Kirsner, 2022; Kenny et al., 2012).

Psychological support involving the selection of appropriate preventive and therapeutic strategies (Spahn, 2011) should be grounded in a thorough assessment conducted using reliable and valid measurement methods. Based on existing multifactor explanatory models of MPA in the psychology of music (e.g., Kenny, 2011; LeBlanc, 1994; Papageorgi et al., 2007), several guidelines can be formulated to inform the selection of tools for measuring MPA in live performance settings:

- The tool should account for all three categories of MPA symptoms: physiological, cognitive, and behavioral.
- Given that performance anxiety is a dynamic emotional state that fluctuates over time, it is important that the instrument is capable of assessing situational MPA at key stages of the performance process—before, during, and after the performance.
- The tool should also allow for the assessment of key situational variables influencing the level of experienced anxiety (e.g., self-efficacy beliefs, González et al., 2018; McCormick and McPherson, 2003; McPherson and McCormick, 2006; Spahn et al., 2021; coping strategies, Biasutti and Concina, 2014; Kaleńska-Rodzaj, 2023).
- It should be concise and feasible for musicians to complete under real performance conditions.
- It must demonstrate strong psychometric properties.

The analysis of English-language tools available for research purposes—such as the Performance Anxiety Inventory (Nagel et al., 1981), the Kenny Music Performance Anxiety Inventory (K-MPAI, Kenny and Osborne, 2006; Kenny, 2023), the Performance Anxiety Inventory for Musicians (PerfAIM, Barbeau, 2011, 2017), and the Young Musicians’ Performance Questionnaire (YMPQ, Papageorgi, 2007) – indicates that all of these instruments primarily assess vulnerability to MPA and the average experience of MPA over a longer period of time, that is, dispositional MPA. This is evident from their theoretical foundations, the way instructions are formulated (e.g., “how you feel generally and how you feel before or during a performance,” K-MPAI; “based on your most recent stressful performance situations, please indicate with a checkmark how much you agree with each statement,” PerfAIM), the content of the items, and the response scale employed (e.g., “always”–“never,” PAI, YMPQ).

In the Polish context, research on music performance anxiety (MPA) has a long and diverse history. One example is the theory of pre-, during and post-performance MPA developed by Kępińska-Welbel (1991), which anticipated similar international conceptualizations far in advance. Another notable approach is the attempt to conceptualize MPA as a mixed, secondary emotion (Kaleńska-Rodzaj, 2020, 2021). However, despite the development of indigenous theoretical perspectives, they have not resulted in the creation of standardized measurement tools. As a consequence, even doctoral dissertations have often involved the development of original pilot scales or the adaptation of existing instruments for research purposes (e.g., K-MPAI, Sadowski, 2020; PAI, Kaleńska, 2010). Over time, several instruments measuring dispositional MPA have been formally adapted into Polish, including the K-MPAI (Kantor-Martynuska and Kenny, 2018), and the adaptation of Steptoe and Fidler’s Self-Statement Scale for measuring MPA coping strategies (Tokarz and Kaleńska, 2005). Currently, the K-MPAI is the most widely used tool in Polish research on MPA, with the PAI being the second most frequently applied instrument.

To sum up, the review of instruments used internationally and in Poland showed that while there are several tools available to measure dispositional MPA — that is, a musician’s general vulnerability to MPA —tools focusing on situational MPA are especially needed and valuable. One particular questionnaire considering situation-related MPA is the Performance-specific Questionnaire for Musicians (PQM).

### 1.1 The Performance-specific Questionnaire for Musicians (PQM) as a tool for assessing music performance anxiety in live performance situations

Recognizing the gap in methods for measuring situational MPA, Spahn and Nusseck developed the Performance-specific Questionnaire for Musicians (PQM) in the German language (“Fragebogen zum Auftritt für Musiker*innen FZAM”; Spahn and Nusseck, 2025). The PQM is designed to assess MPA specifically in live performance situations. This self-report instrument addresses particular symptoms of MPA—physiological, cognitive, and behavioral—as well as variables most strongly associated with the level of experienced anxiety: self-efficacy (SE, defined as one’s confidence in performing) and functional coping (strategies employed to manage MPA). The questionnaire retrospectively covers the time before and during the performance and also includes a post-performance section, requiring completion immediately following the event.

The structure of the PQM is clear and user-friendly, designed to minimize respondent burden. The tool uses the same set of 11 statements (presented in random order with minor modifications relating to the temporal context of the performance) to retrospectively describe feelings *Before* (anticipation) and *During* the performance (execution). The *After* scale (post-performance) includes 11 items focusing on self-evaluation of the performance experience, thus differing in content from the previous two sections. Each of the three subscales (*Symptoms of MPA*, *Self-efficacy*, and *Functional coping*) is brief, and comprises three to four items. Participants rate 33 statements in reference to the performance they have just completed, using a 5-point Likert scale from 1 - “not true at all” to 5 - “very true.”

Additionally, the PQM includes a section with seven items assessing the subjective quality of the performance, enabling the investigation of the influence of MPA symptoms, SE, and coping strategies on the self-rated performance quality and thus capturing the functional significance of the measured variables. These music-related aspects are rated on a 6-point scale ranging from 1 - “very poor” to 6 - “excellent.”

The PQM was first developed for an intervention study with a German sample of music students to investigate effects of a simulated audition on situational MPA (Spahn et al., 2016). It was then extended and validated as part of a medical dissertation (Biwer, 2015). Cronbach’s alpha coefficients for the subscales across the three temporal contexts were as follows: for *Symptoms of MPA*—before 0.81, during 0.83, after 0.67; for *Self-Efficacy*—before 0.71, during 0.77, after 0.83, and for *Functional Coping with MPA*—before 0.73, during 0.80, after 0.66. Cronbach’s alpha for the additional seven-item scale assessing self-perceived musical quality was 0.77 (Spahn and Nusseck, 2025).

Further validation studies were conducted on samples of 363 adult orchestral musicians (Spahn et al., 2021) and 67 young amateur musicians (Spahn et al., 2023) indicate satisfactory psychometric properties. In these studies, the PQM has been used and correlated with other standard questionnaires. The correlation with the Flow Short Scale total scores (Rheinberg et al., 2003) revealed expected relationships with the PQM (Spahn et al., 2021). The subscale *Symptoms of MPA* was negatively correlated with the overall flow score in all temporal contexts and the *Functional Coping* as well as the *Self-Efficacy* subscales showed positive correlations. Comparisons between the PQM and the general disposition of MPA measured with the K-MPAI (Kenny, 2009) found low positive correlations between the subscale *Symptoms of MPA* with the total K-MPAI score before and after the performance and negative correlations with *Functional Coping* after performance. The subscale *Self-Efficacy* was negatively correlated with the K-MPAI across all temporal contexts (Spahn and Nusseck, 2025).

In light of the discussed requirements for live performance MPA assessment tools, the PQM stands out as a promising candidate to fulfil these criteria. Given its numerous advantages, an adaptation of the PQM for Polish conditions has been undertaken, enabling at the same time Polish researchers to investigate situational MPA in live performance contexts.

### 1.2 Purpose of study

The primary aim of the present study was to prepare a Polish version of the PQM scale (German version, Spahn and Nusseck, 2025) and to examine its psychometric properties, including factor structure and external reliability.

The selection of instruments for assessing external validity was based on three criteria: (1) the use of tools with an established position in research on Polish-speaking populations and (2) proven psychometric quality, and (3) the measurement of constructs conceptually related to those assessed by the PQM. The Performance Anxiety Inventory (PAI) was selected as the counterpart to the Symptoms of MPA scale, the Flow Short Scale (FSS) as the counterpart to the Functional Coping scale, and the General Self-Efficacy Scale (GSES) as the counterpart to the Self-Efficacy scale. The use of the FSS, rather than instruments designed specifically to measure coping strategies, allows for direct comparison with results obtained in studies on the original PQM (Spahn and Nusseck, 2025; Spahn et al., 2021) and takes into account strategies beneficial for reducing MPA. The PAI was chosen over the K-MPAI due to its shorter format and ease of administration in performance situations. All questionnaires were used in the Polish version. The following section outlines the process of developing the Polish adaptation of the PQM.

## 2 Method

### 2.1 Participants

The original research sample consisted of 289 participants; however, to minimize the risk of including responses that were provided either too hastily or with excessive delay, a response time criterion ranging from 6 to 30 min was applied. This procedure was intended to enhance data quality and ensure that the final analyses were based on valid and reliable responses.

The final sample comprised 256 Polish classical musicians, including 71 musicians aged 18–72 (*M* = 29.91, SD = 12.26) and 185 musicians (79.4%) in the 18–34 age range. There were 178 women (69.8%), 74 men (29.0%), two participants identifying as a different gender (0.8%). There were more instrumentalists (*N* = 172, 67.5%) than vocalists (*N* = 83, 32.5%). The sample included individuals with different roles within the performance: ensemble musicians (*N* = 208, 81.6%), soloists (*N* = 25, 9.8%), and musicians performing both roles (*N* = 22, 8.6%). Most participants were orchestra and choir musicians at the professional level (three music academy students orchestras and five choirs, one opera orchestra and choir) and semi-professional level (one university orchestras and three choirs).

### 2.2 Measures

*Performance-specific Questionnaire for Musicians* (PQM; Spahn and Nusseck, 2025; Spahn et al., 2016, 2021, 2023) is used to assess situational MPA in live performance situations. The questionnaire was described in the previous section. All original items were first translated into Polish by a bilingual professional speaker. The resulting version was reviewed by two bilingual professional musicians to identify potential mistranslations and misunderstandings. Small adaptations of the translation were made in the items 4 and 7 to ensure that the intended meaning of the original items was preserved.

*The Performance Anxiety Inventory* (PAI; Nagel et al., 1981) is used to assess somatic, cognitive, and behavioral symptoms of MPA. The Polish version of the PAI (Kaleńska, 2010) comprises 20 items, each rated on a 4-point Likert scale measuring frequency (1 - “almost never” to 4 - “almost always”). Respondents indicate how they generally feel when performing in front of an audience. Total scores range from 20 to 80, with higher scores reflecting greater vulnerability to MPA. Cronbach’s alpha for scale reliability in two samples of violinists was 0.90 and 0.92.

*The General Self-Efficacy Scale* (GSES; Schwarzer and Jerusalem, 1995) is used to assess individuals’ belief in their ability to manage difficult situations and cope with adversity. The Polish version of the GSES (Jurczyński, 2000) comprises 10 items each rated on a 4-point Likert scale (1 – “not at all true” to 4 - “exactly true”). Total scores range from 10 to 40, with higher scores indicating a stronger sense of self-efficacy. Cronbach’s alpha for scale reliability is 0.85.

*The Flow Short-Scale* (FSS; Rheinberg et al., 2003) is used to assess self-perceived flow on a continuous scale, assessing retrospectively the experience of flow during a recently completed task. In this study, it refers specifically to the participants’ most recent musical performance, thus capturing situational flow. The Polish version of FSS (Wojtasiński et al., 2024) comprises 10 items each rated on a on a 7-point scale (1 - “strongly disagree” to 7 - “strongly agree”). All items contribute to the Total Flow Score, with higher values indicating a greater overall flow experience (Cronbach’s alpha 0.89). The questionnaire includes two subscales: (1) Fluency of the Performance, reflecting perceived automated processing of the activity, with higher scores indicating a more fluent performance (Cronbach’s alpha 0.87), and (2) Absorption in the Activity, reflecting the extent to which participants lose awareness of time while engaged in the task, with higher scores indicating deeper absorption (Cronbach’s alpha 0.75).

### 2.3 Procedure

Participation in the research project was voluntary and anonymous, with written informed consent obtained from all participants. During the final rehearsal prior to the concert, participants were informed about the study and the procedure for participation. Immediately after the concert performance, the musicians completed self-report inventories. The concerts were held in public venues, with audience sizes ranging from 50 to 300 people. After the performance, participants scanned a QR code and completed the inventories electronically, allowing them to fill in the questionnaires conveniently in a backstage setting. Each participant received the equivalent of a €7 voucher as reward for their participation. The study was approved by the Ethical Committee of the University of the National Education Commission in Cracow.

### 2.4 Data analysis

All statistical analyses were conducted using JASP software (version 0.19.1.0), which provides a comprehensive and user-friendly environment for advanced statistical modeling. In the first step the program was employed to perform confirmatory factor analysis (CFA) and factor loadings in order to evaluate the factorial structure of the measured constructs. In the CFA, the maximum likelihood (ML) estimator was employed, as it is widely regarded as the most appropriate method for obtaining reliable parameter estimates and model fit indices under conditions of multivariate normality. In addition, reliability indices were computed, with Cronbach’s alpha serving as an indicator of the internal consistency. To further assess external validity, correlation matrices were generated and analyzed, allowing for the examination of the relationships between the studied variables and theoretically relevant external criteria. The use of JASP ensured both methodological transparency and reproducibility of the statistical procedures.

## 3 Results

### 3.1 Structure verification

In the confirmatory factor analysis (CFA), the factorial structure of the subscales within the PQM questionnaire was systematically examined for the *Before*, *During*, and *After* scales. The analysis was conducted with reference to the theoretical framework and measurement model proposed in the original version of the PQM (Spahn and Nusseck, 2025).

In evaluating model fit, the acceptance criteria were established based on commonly cited recommendations in the literature. Specifically, the ratio of *χ*^2^ to degrees of freedom (*χ*^2^/df) with a recommended value below 5 was assessed following the guidelines proposed by Kline (2016). The cut-off values for a good model fit with recommended values of CFI > 0.9, TLI > 0.9, RMSEA < 0.08, and SRMR < 0.06 were adopted in accordance with Hu and Bentler (1999). The results for the *Before* and *During* scales indicated good levels of model fit for the original factorial structure. However, for the *After* scale, the original structure did not meet the acceptance thresholds, necessitating an optimization of the subscale items. With very low factor loadings between 0.26 and 0.28 in the EFA, items 27, 28, and 32 were removed from the model. Following this optimization, the fit indices reached satisfactory levels, thereby supporting the adequacy of the adjusted factorial structure. The detailed results are presented in Table 1.

**Table 1:** Final CFA fit measures for all PQM scales without items 27, 28 and 32.

Scale	χ^2^//df	CFI	TLI	SRMR	RMSEA	RMSEA 90% CI	
						Lower	Upper
Before	1.675	0.974	0.964	0.035	0.051	0.029	0.072
During	2.466	0.961	0.948	0.038	0.076	0.057	0.095
After	2.790	0.961	0.936	0.042	0.084	0.056	0.113

**Figure 1:**
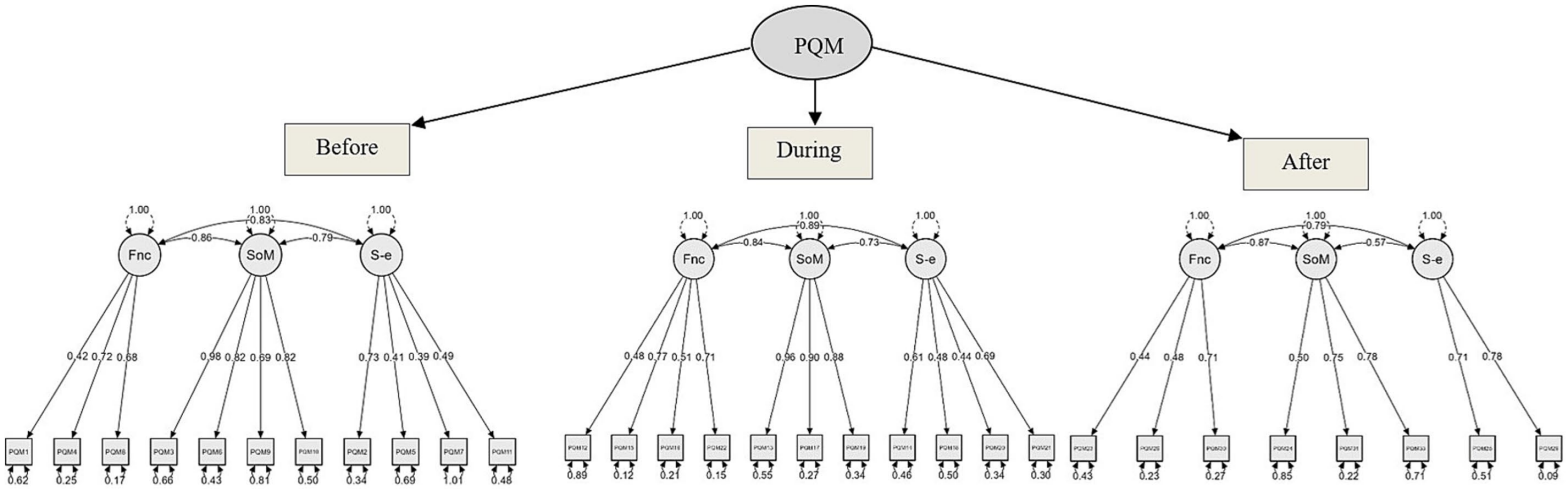
Final structure of PQM scales and subscales in the time points before, during and after the performance (Fnc, Functional coping; SoM, Symptoms of MPA; S-e, Self-efficacy).

The final structure of all scales is presented below in the form of a model plots (Figure 1) providing a graphical representation of the confirmed factorial solution. The individual item-scale connection for each PQM scale in the time points before, during and after the performance are shown by the loading estimates and the error variance estimates of each item.

### 3.2 Internal consistency

The lowest factor loading was observed for item PQM7, with a value of 0.392. Although a commonly recommended threshold for acceptable factor loadings is > 0.40 (Brown and Ryan, 2003), the value obtained was only marginally below this criterion. Considering the close proximity to the recommended cut-off, the intention to preserve the original factorial structure of the PQM, and the relatively large sample size, the decision was made to retain this item in the questionnaire. The Table 2 presents the factor loadings for all items included in the PQM questionnaire, providing a comprehensive overview of the measurement model across the assessed scales.

**Table 2:** Factor loadings for the PQM scales.

Subscale	Item	Loading	Std. Error	*z*-value	*p*	95% Confidence interval	
						Lower	Upper
Factor loadings for *Before* PQM scale							
Functional coping	PQM1	0.418	0.056	7.489	< 0.001	0.309	0.527
	PQM4	0.720	0.048	15.095	< 0.001	0.626	0.813
	PQM8	0.682	0.043	15.994	< 0.001	0.598	0.765
Symptoms of MPA	PQM3	0.977	0.071	13.714	< 0.001	0.837	1.116
	PQM6	0.821	0.059	13.996	< 0.001	0.706	0.936
	PQM9	0.692	0.068	10.138	< 0.001	0.558	0.826
	PQM10	0.822	0.061	13.451	< 0.001	0.702	0.942
Self-efficacy	PQM2	0.730	0.057	12.871	< 0.001	0.619	0.841
	PQM5	0.413	0.062	6.713	< 0.001	0.293	0.534
	PQM7	0.393	0.073	5.378	< 0.001	0.250	0.536
	PQM11	0.492	0.054	9.095	< 0.001	0.386	0.598
Factor loadings for *During* PQM scale							
Functional coping	PQM12	0.476	0.065	7.335	< 0.001	0.349	0.603
	PQM15	0.772	0.041	18.668	< 0.001	0.691	0.853
	PQM16	0.510	0.038	13.553	< 0.001	0.436	0.583
	PQM22	0.711	0.041	17.496	< 0.001	0.632	0.791
Symptoms of MPA	PQM13	0.958	0.066	14.557	< 0.001	0.829	1.087
	PQM17	0.904	0.054	16.711	< 0.001	0.798	1.010
	PQM19	0.876	0.056	15.644	< 0.001	0.766	0.986
Self-efficacy	PQM14	0.615	0.054	11.308	< 0.001	0.508	0.721
	PQM18	0.476	0.053	8.944	< 0.001	0.371	0.580
	PQM20	0.441	0.044	9.909	< 0.001	0.353	0.528
	PQM21	0.686	0.050	13.653	< 0.001	0.588	0.785
Factor loadings for *After* PQM scale							
Functional coping	PQM23	0.438	0.049	8.918	< 0.001	0.342	0.535
	PQM26	0.484	0.039	12.341	< 0.001	0.407	0.561
	PQM30	0.710	0.050	14.333	< 0.001	0.613	0.807
Symptoms of MPA	PQM24	0.505	0.068	7.446	< 0.001	0.372	0.638
	PQM31	0.747	0.051	14.655	< 0.001	0.647	0.847
	PQM33	0.785	0.070	11.248	< 0.001	0.648	0.921
Self-efficacy	PQM25	0.706	0.061	11.580	< 0.001	0.587	0.826
	PQM29	0.784	0.050	15.722	< 0.001	0.686	0.881

### 3.3 Reliability

The reliability coefficients of the subscales ranged from Cronbach’s alpha 0.63 to 0.87. The lowest value was observed for the Self-efficacy subscale within the *Before* scale. Reliability was also assessed for the Performance Quality scale (PQM_Q), which serves as a supplementary measure of the musician’s subjective evaluation of performance quality. Nevertheless, all obtained results were considered acceptable, supporting the internal consistency of the PQM subscales. The results are presented in Table 3.

**Table 3:** Cronbach’s alpha values for all PQM subscales.

Subscale	Before	During	After
Functional coping	0.74	0.80	0.73
Symptoms of MPA	0.82	0.87	0.70
Self-efficacy	0.63	0.75	0.79

The reliability coefficient of the Performance Quality scale was Cronbach’s alpha 0.85.

### 3.4 Theoretical validity

Theoretical validity was examined through correlations with the PAI, FSS, and GSES questionnaires, as well as by analyzing associations between the PQM subscales and the performance quality scale (PQM_Q). Spearman’s *ρ* coefficient was applied due to the violation of the normality assumption in some items. All obtained correlations were statistically significant at *p* < 0.001. As hypothesized, total scores of the FSS and GSES questionnaires correlated positively with the *Functional coping* and *Self-efficacy* subscales, and negatively with the *Symptoms of MPA* subscale. Conversely, the PAI total score correlated negatively with the *Functional coping* and *Self-efficacy* subscales, and positively with the *Symptoms of MPA* subscale. These hypotheses were supported by the data, with observed correlations ranging from 0.247 to 0.566.

Regarding correlations with the performance quality scale (PQM_Q), positive associations were expected for the *Functional coping* and *Self-efficacy* subscales, and negative associations for the *Symptoms of MPA* subscale. These hypotheses were supported by the data, with observed correlations ranging from 0.304 to 0.547. The detailed results are presented in Table 4.

**Table 4:** Spearman’s ρ coefficient – associations between the PQM subscales and musicians results in other questionnaires.

Variable	1	2	3	4	5	6	7	8	9
1. Functional Coping (B)	—								
2. Symptoms of MPA (B)	−0.650***	—							
3. Self-Efficacy (B)	0.530***	−0.480***	—						
4. Functional Coping (D)	0.732***	−0.587***	0.554***	—					
5. Symptoms of MPA (D)	−0.676***	0.841***	−0.493***	−0.651***	—				
6. Self-Efficacy (D)	0.592***	−0.457***	0.751***	0.657***	−0.542***	—			
7. Functional Coping (A)	0.472***	−0.341***	0.654***	0.564***	−0.398***	0.761***	—		
8. Symptoms of MPA (A)	−0.440***	0.427***	−0.442***	−0.486***	0.453***	−0.520***	−0.545***	—	
9. Self-Efficacy (A)	0.429***	−0.298***	0.557***	0.464***	−0.365***	0.569***	0.564***	−0.273***	—
10. Performance Quality	0.412***	−0.304***	0.502***	0.408***	−0.381***	0.547***	0.456***	−0.386***	0.332***
11. PAI total score	−0.339***	0.446***	−0.352***	−0.393***	0.456***	−0.426***	−0.304***	0.316***	−0.247***
12. FSS total score	0.401***	−0.303***	0.472***	0.457***	−0.394***	0.566***	0.513***	−0.353***	0.380***
13. GSES total score	0.424***	−0.305***	0.391***	0.393***	−0.345***	0.469***	0.414***	−0.368***	0.359***

(B)- Before scale, (D) – During scale, (A) – After scale, PAI, Performance Anxiety Questionnaire; FSS, Flow Short Scale; GSES; General Self-Efficacy Scale; * *p* < 0.05, ** *p* < 0.01, *** *p* < 0.001.

It is worth to mention, that the intercorrelations among the PQM subscales ranged from moderate to strong (0.273–0.841), indicating that the subscales are relatively strongly related, which may be regarded as a positive indicator of the overall reliability of the questionnaire.

## 4 Discussion

The aim of the present study was the Polish adaptation and validation of the PQM. The results of the confirmatory factor analysis confirmed the stability of the factor structure of the original PQM: three subscales (Symptoms of MPA, Functional Coping, and Self-Efficacy) assessed across three temporal conditions (Before, During, and After). The Before and During scales preserved the identical number and content of items as in the original PQM. The After scale, however, did not exhibit comparable consistency. Yet, once items 27, 28, and 32 were removed due to low factor loadings, model - data fit indices reached satisfactory levels. It is worth noting that item 27 had also been excluded from the original PQM for the same reason (Spahn et al., 2016).

An analysis of factor loadings across subscales indicated strong coherence for Symptoms of MPA and Functional Coping, but weaker consistency for Self-Efficacy. This may be attributable to the increased heterogeneity of item content following the translation into Polish. For example, item 5 was translated in a way that may have encouraged respondents to interpret it as referring to their ability to imagine a satisfied audience, rather than to their belief in whether they could, in the sense of self-efficacy, do so. This subtle linguistic shift - from the original “I could imagine…” toward a construction closer to “I was able to imagine…” - likely altered the cognitive focus of the item. As a result, the Polish version may have been perceived as assessing a cognitive skill or imaginative capacity rather than an efficacy judgment. A similar semantic drift appeared in the translation of item 7, which refers to anticipation before performance. Whereas the original phrase “I’m looking forward…” emphasizes positive, excited anticipation, the Polish equivalent conveys impatience with a potentially negative emotional load (“I’m waiting impatiently…”). Such semantic and affective discrepancies may have contributed to lower internal consistency by reducing the homogeneity of items intended to measure self-efficacy.

The findings support the theoretical validity of the Polish PQM. Correlations among the subscales followed the expected pattern: Symptoms of MPA correlated negatively with Self-Efficacy and Functional Coping, while the latter two were positively associated with each other. All three subscales demonstrated high correlations across the three temporal scales (Before, During, After). Moreover, associations between PQM subscales and external measures of related constructs aligned with theoretical expectations. Specifically, Symptoms of MPA correlated positively with performance anxiety vulnerability as measured by the PAI. The study with the correlation between Symptoms of MPA and MPA vulnerability as measured by the K-MPAI found relatively low and nonsignificant correlations in the During condition (Spahn et al., 2023). By contrast, the present study yielded higher and statistically significant associations in each temporal condition, likely due to the use of the PAI, which focuses exclusively on somatic, cognitive, and behavioral symptoms of MPA. Correlations of Functional Coping with the FSS total score were positive, moderate in size, and highly similar to those reported by Spahn et al. (2021). A novel aspect of the present study was the use of the General Self-Efficacy Scale (GSES) to assess general efficacy beliefs, which showed a moderate positive correlation with the PQM Self-Efficacy subscale. Regarding performance quality as the outcome variable, the expected associations were observed: higher Self-Efficacy and Functional Coping scores, and lower Symptoms of MPA scores, predicted more favorable self-evaluations of performance quality.

The correlation between Functional Coping and Self-Efficacy was moderately high. Content analysis of items suggests some overlap: both subscales capture self-efficacy beliefs, albeit in different domains. Functional Coping reflects efficacy beliefs specifically in emotion regulation, whereas Self-Efficacy relates more directly to preparation and performance skills. Considering both types of self-efficacy beliefs in live performance research is particularly valuable, as musicians’ satisfaction with performance encompasses not only quality of execution but also well-being on stage.

### 4.1 Limitations and future directions

Despite the strong model fit indices observed for the Polish PQM, several limitations should be noted. First, the generalizability of findings is constrained by the characteristics of the sample, which consisted of young adult ensemble musicians who voluntarily participated in the study immediately after a concert. Recruiting musicians at this time point – when relief after performance is particularly salient – proved challenging and may have introduced selection bias, including with respect to participants’ motivation. When employing the PQM in future studies, it would be worthwhile to also assess intrinsic motivation with complementary measures to provide a more comprehensive account of the phenomenon. Future research should also examine the applicability of the Polish PQM across diverse musician populations and contexts to confirm its broader validity and utility. One potential direction for extending the study on the Polish sample could involve comparing the mean scores obtained on the individual subscales of the Before, During, and After scales, in a manner consistent with the procedure employed in the study by Spahn et al. (2021).

Importantly, the availability of a multilingual method for assessing flow in performance contexts creates opportunities for cross-national comparisons with reduced risk of methodological bias. Such work will help advance research on the situational dynamics of MPA.

With respect to prevention and intervention, the PQM appears to be a valuable diagnostic tool for assessing musicians’ well-being during live performance. Its structure accounts for both vulnerability factors (Symptoms of MPA) and protective factors (Coping and Self-Efficacy), thereby providing a practical foundation for developing tailored psychological support programs for specific groups of performers.

## 5 Conclusion

The Polish version of the Performance-specific Questionnaire for Musicians (PQM) consists of 37 items (with 7 items of Performance Quality section). It captures three categories of MPA symptoms (physiological, cognitive, behavioral) and two key situational variables influencing anxiety levels (Functional Coping and Self-Efficacy). The measure assesses their dynamic interplay across three temporal conditions (Before, During, After) and their impact on performance (self-evaluation of performance quality). The scale is brief and feasible to administer under real performance conditions. With its sound psychometric properties, the Polish PQM represents a promising tool for research on MPA and its antecedents in live performance, as well as for psychological interventions designed for musicians.

## Data Availability

The raw data supporting the conclusions of this article will be made available by the authors, without undue reservation.
